# A Method and On-Line Tool for Maximum Likelihood Calibration of Immunoblots and Other Measurements That Are Quantified in Batches

**DOI:** 10.1371/journal.pone.0149575

**Published:** 2016-02-23

**Authors:** Steven S. Andrews, Suzannah Rutherford

**Affiliations:** 1 Basic Sciences Division, Fred Hutchinson Cancer Research Center, Seattle, WA, United States of America; 2 Department of Physics, Seattle University, Seattle, WA, United States of America; University of California, Davis, UNITED STATES

## Abstract

Experimental measurements require calibration to transform measured signals into physically meaningful values. The conventional approach has two steps: the experimenter deduces a conversion function using measurements on standards and then calibrates (or normalizes) measurements on unknown samples with this function. The deduction of the conversion function from only the standard measurements causes the results to be quite sensitive to experimental noise. It also implies that any data collected without reliable standards must be discarded. Here we show that a “1-step calibration method” reduces these problems for the common situation in which samples are measured in batches, where a batch could be an immunoblot (Western blot), an enzyme-linked immunosorbent assay (ELISA), a sequence of spectra, or a microarray, provided that some sample measurements are replicated across multiple batches. The 1-step method computes all calibration results iteratively from all measurements. It returns the most probable values for the sample compositions under the assumptions of a statistical model, making them the maximum likelihood predictors. It is less sensitive to measurement error on standards and enables use of some batches that do not include standards. In direct comparison of both real and simulated immunoblot data, the 1-step method consistently exhibited smaller errors than the conventional “2-step” method. These results suggest that the 1-step method is likely to be most useful for cases where experimenters want to analyze existing data that are missing some standard measurements and where experimenters want to extract the best results possible from their data. Open source software for both methods is available for download or on-line use.

## Introduction

Nearly every quantitative experiment requires calibration—the mathematical conversion of raw measurements into physically meaningful values. For example, calibration of immunoblot (Western blot) data converts the intensities of protein bands that are detectable on a blot into the concentrations of proteins that were present in the original samples. Although many scientists take calibration for granted, we show here that conventional approaches are not particularly accurate, causing them to lose some of the information that is carried by valuable measurement data. As a result, they provide a less than optimal estimate of the true sample values. To address this, we developed an approach that systematically exploits all available information in the data and returns the most accurate results possible within the constraints of a statistical model.

The classical solution to the linear calibration problem [1–4] is a two step process: first, during the calibration step, measurements on known samples, “standards,” are used to deduce a conversion function. Then, during the prediction step, the conversion function is used to convert measurements on unknown samples to physical quantities. For example, suppose a chemist uses an instrument whose response is linear in the amount of protein, chemical, or other analyte in a sample. This means that an instrument measurement, *y*, is related to the amount of analyte, *x*, according to the response function

y=α+βx+ε. (1)

The *α* and *β* parameters are instrument-specific sensitivity coefficients and *ε* represents random measurement noise. In the calibration step, the chemist estimates the *α* and *β* sensitivity coefficients, yielding *a* and *b* respectively, by measuring several standards with known compositions and fitting the resulting data with Eq 1 using linear regression. Substituting the regression results into Eq 1 and solving for *x* yields the conversion function

$$x≃\frac{y-a}{b}.$$ (2)

In the prediction step, the chemist measures samples of unknown composition on the same instrument and inserts the measurements into Eq 2. This yields the sample analyte amounts.

In this example, note that errors in the standard measurements lead directly to errors in the sensitivity coefficient estimates. From there, they lead to errors in the computed analyte amounts. For this reason, it is good practice to measure standards repeatedly because this reduces the effects of their errors through averaging. More standard measurements also help because they can enable the experimenter to test the instrument (or method) response linearity (e.g. see ref. [5]). However, the number of standard measurements is usually limited by several factors. First, each standard measurement costs time and materials. Also, standard measurements often replace the opportunity to measure unknown samples; for example, protein electrophoresis gels have a fixed number of lanes, so lanes that are used for standards cannot be used for unknown samples. Additionally, experimental mistakes or artifacts may make some standard measurements invalid.

Calibration often needs to be performed repeatedly. For example, many experimental methods analyze samples in groups in which the sensitivity is the same for all measurements within a group but different for measurements in different groups (e.g. immunoblots and ELISA assays). Multiple calibrations are also required when one has many instruments that have different sensitivities. Additionally, most instrument sensitivities “drift” over time, necessitating periodic re-calibration (e.g. spectrometers and chromatographs). For convenience, we call all of these situations “batch-analyses,” defining a batch as any collection of measurements for which the sensitivities can be considered to be constant. By implication, each batch requires its own calibration.

We show here that calibrating each batch independently of the others, which is typical, is not the best approach because the results are very sensitive to errors in the standard measurements. However, if sample replicates are spread across different batches, then calibrating all batches in a simultaneous analysis can substantially reduce the effects of measurement noise. In brief, our approach is to fit a statistical model to all of the data in a single step, finding both the instrument sensitivities and analyte amounts that best agree with all of the measurements. In other words, we cross-calibrate each batch against every other one. We call this the 1-step method, in contrast to the conventional 2-step method. The principle advantage of the 1-step method is that it makes calibration less sensitive to individual standard measurements. This often enables the use of batches that did not include any standards and it also enables the detection of errors in standard measurements. The results of the 1-step method are the maximum likelihood predictors, meaning that they are the results that are most probable within the assumptions of a statistical model.

We developed the 1-step calibration method to analyze data that we recently collected on proteins in mouse skin tumors. Our goal was to compare the relative levels of each of 7 different proteins (CypA, Hsp90, Hsp70, Hsc70, P53, Raf, and pERK) in 230 precancerous and cancerous mouse skin tumors using quantitative immunoblotting methods [6–10]. In brief, tumor extracts (replicated, pre-mixed with denaturing SDS-sample buffer, and stored at -80 C in small aliquots to maintain their integrity) were run on polyacrylamide gels (SDS-PAGE) to separate proteins by size and charge, followed by their transfer to nitrocellulose membranes. To individually probe query proteins of different molecular weights, the membranes were cut into horizontal strips bracketing size ranges determined by visible molecular weight standards that were run with each gel. Each strip, usually containing just one, or at most two query proteins of close molecular weight, was probed with the appropriate primary antibody (Spratt et al., in preparation). This was followed by incubation with secondary antibodies linked to an infrared fluorophore using the LICOR fluorescent Western blot detection system [11,12]. This method assured that signal intensity was linear within a large dynamic range.

Calibrating these data was challenging for several reasons. First, immunoblotting is inherently imprecise. Indeed, all of the samples in our study, including those for standards, exhibited substantial measurement error (after calibration, our average CV was 41%). For this reason, we analyzed each sample multiple times on different blots so that we could reduce the effects of measurement noise through averaging. In total, we analyzed 230 tumor extracts on 117 immunoblots, each of which held up to 20 lanes (1510 replicated samples total, average of 6 replicates/sample). Secondly, one cannot directly compare fluorescence measurements between different blots because each blot’s sensitivity is strongly affected by minor experimental differences [9]. As a result, each blot needed to be treated as its own batch, with its own batch-specific sensitivity (calibration showed that they varied 27-fold between least and most sensitive). Finally, we could not use internal standards in this investigation (see [6]), which in this case would be naturally expressed proteins that are expected to have nearly constant concentrations such as the products of housekeeping genes, because tumors are very heterogeneous. As a result, we had to use a separate external standard, which was then subject to independent measurement errors. We created our standard by pooling several samples together to produce a single sample that included all of our proteins of interest [13].

Our 1-step calibration method is sufficiently straightforward that it would be surprising if some version of it has not been used previously (see related work in [14–16]). In particular, many biologists have likely used common samples to connect different batches in an ad hoc manner. Our method applies this logic systematically over an entire data set, simultaneously finding the best solution given all of the data.

Our 1-step calibration method is distinct from several other modifications to the classic calibration problem. Of particular note, Krutchkoff showed, nearly 50 years ago, that it can be better to fit the experimental results for the standard using the conversion function (Eq 2), rather than with the response function (Eq 1), which is called the inverse approach [17,18]. This led to an active debate about the relative merits of the two methods, along with the development of inverse regression methods [2,4,19]. From our reading of the literature, this debate appears to have largely ended by now, although without a clear winner. Other modifications to the classic calibration problem include Bayesian [20] and non-parametric [3,21] methods. Bayesian methods are particularly helpful when the instrument is relatively insensitive to analyte variation (i.e. *β* is small) and the non-parametric methods when the measurement errors are substantially non-normally distributed. Finally, bootstrapping methods [22,23] can provide more accurate confidence intervals for the results, particularly for multivariate problems. In contrast to these developments, our 1-step approach follows the style of the classic calibration approach, keeping the linear statistical model and the least squares fitting approaches. It extends the classic calibration approach to optimally account for multiple batches.

## Results

### Definitions and model

Extending the analytical chemistry example given above, consider the situation in which one is quantifying the amount of an analyte in each of many samples, where a sample is simply some quantity of material. Assume this work is performed in batches, where a batch is a collection of measurements for which the instrument (or experimental method) sensitivity can be assumed to be constant. Additionally, assume that one or more standards are included in the analysis, where the standards already have well characterized analyte amounts. If such a standard is not available, then one simply assigns the role of the standard to one of the unknown samples and measures the other analyte amounts relative to that one. Our case followed this situation reasonably closely: the different mouse tissue extracts were our samples, the measured protein species in these samples were our analytes, the immunoblot gels were our batches, and the pooled sample served as our standard. This situation generalizes to many other calibration problems, too.

Assume that the following statistical model accurately represents the experimental data:

$$y_{ijk}=\alpha_{i}+\beta_{i}x_{j}+\varepsilon_{ijk}.$$ (3)

On the left side of the equation, each *y*_*ijk*_ value represents a single measurement, where *i* is the batch number, *j* is the sample number, and *k* distinguishes between multiple measurements of a particular sample that are within a single batch. Every measurement can be assigned a unique set of *i*, *j*, and *k* subscripts and so can be identified in this way. However, this does not necessarily imply that every sample was measured in every batch. To the contrary, most samples are likely to have been measured only a few times total in the entire experiment, making the *y*_*ijk*_ values a relatively sparse dataset (e.g. we had 230 total samples but only analyzed up to 20 at a time on any given immunoblot). On the right side of the equation, *α*_*i*_ and *β*_*i*_ are batch-specific sensitivity coefficients, *x*_*j*_ is the amount of analyte in sample *j*, and *ε*_*ijk*_ is the measurement error that arose in the *k*’th measurement of sample *j* in batch *i*. Assume that this error is normally distributed with mean of zero and standard deviation of *σ*, and that it is independent between measurements. This statistical model is very simple and builds upon conventional assumptions (including, importantly, that measurements depend linearly upon analyte amounts). It was also appropriate for our work because we confirmed that our immunoblot detection was linear in antigen amounts (and see [12]) and our tests of measurement repeatability showed reasonably independent and normally distributed errors (we found that the distribution of squared differences between repeated measurements of the same samples on the same blots was reasonably exponential, as one would anticipate for normally distributed errors). Table 1 summarizes the nomenclature introduced here.

**Table 1:** Data analysis nomenclature.

**Roman symbols**	
*a*_*i*_, *b*_*i*_	estimates of batch sensitivity parameters
*i*, *N*_*B*_	batch index and number of batches
*j*, *N*_*S*_	sample index and number of samples
*k*	measurement index within a specific batch and sample
*n*_*i*,*j*_	number of measurements of sample *j* in batch *i* (replacing *i* or *j* with “All” denotes all batches or samples)
*SD*_*j*_, *SE*_*j*_	standard deviation (data variability) and standard error (precision of estimate of the mean) of measurements of analyte amount *j*
*T*	list of standards
*x*_*j*_	analyte amount in sample *j*
*y*_*i*,*j*,*k*_	measurement value for measurement *k* of sample *j* in batch *i*
**Greek symbols**	
*α*_*i*_, *β*_*i*_	true batch sensitivity parameters
*ε*_*i*,*j*,*k*_	measurement error for a specific measurement
*σ*	standard deviation of measurement noise
*χ*^2^	goodness of fit parameter

The primary data analysis goal, typically, is to estimate the analyte amounts, *x*_*j*_, and their confidence intervals. Below, we also solve for the sensitivity coefficients, *a*_*i*_ and *b*_*i*_, which can enable one to calibrate any new measurements that were not included in the original data. We also find the measurement standard deviation, *σ*, which can be helpful for improving the measurement technique and for identifying any outlier data points.

### The 2-step method

**Fig 1:**
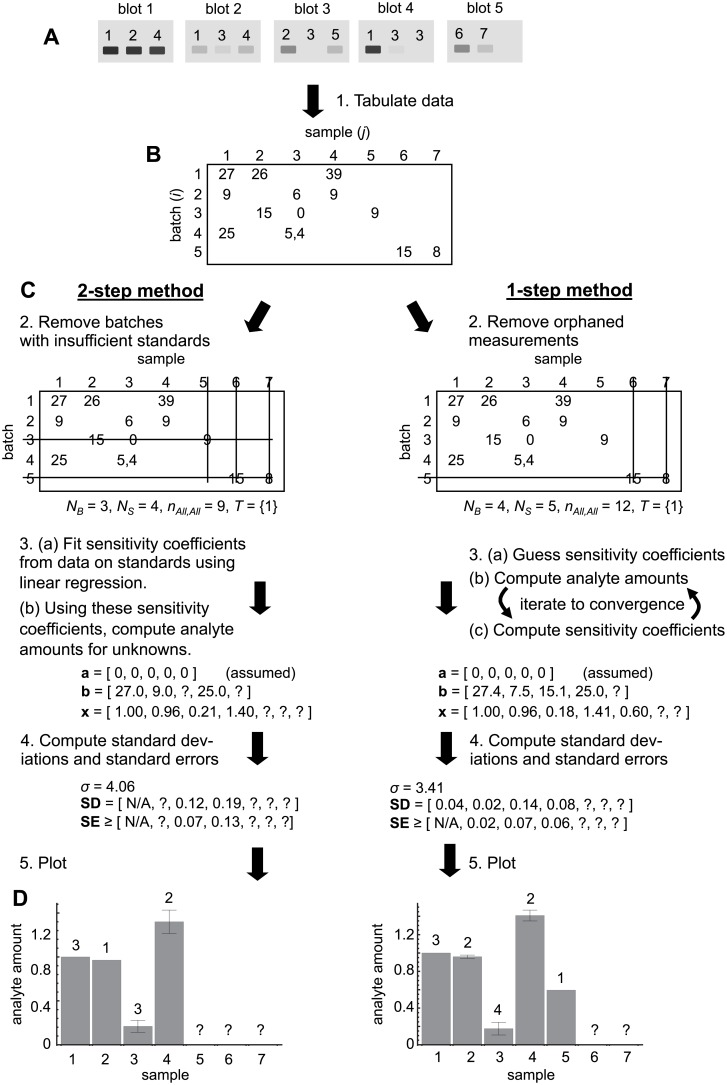
Comparison of workflow for 2-step and 1-step calibration methods, illustrated for calibrating band intensities on immunoblots. A. Illustration of samples 1 (the standard) through 7, run on 5 different immunoblots with variable replication. The band intensities shown depend on the sample, blot, and experimental noise. B. Tabulated data showing assigned band intensities for each sample and blot. C. Direct comparison of the conventional 2-step calibration method (left) with the 1-step calibration method (right). D. Plots of the calibrated estimates of analyte amounts in each sample using the different methods. Error bars represent the standard error of the mean (precision of estimate) and numbers above the bars represent the number of calibrated measurements of each sample.

We present the conventional 2-step calibration method, focusing on its application to samples that are measured in batches, to introduce our mathematical notation in a setting that may be familiar and to show some aspects of the method that are widely overlooked. The left side of Fig 1 illustrates the 2-step method.

#### (1) Tabulate data

The measurements need to be tabulated, putting each sample in a separate column and each batch in a separate row. Each table site has as many entries as there are measurements for that specific sample and batch, which may be zero, one, or more than one.

#### (2) Remove batches with insufficient standards

To enable calibration, each batch needs to include at least as many different standard measurements as there are unknown sensitivity coefficients (because of the linear algebra result that one needs at least *n* equations to solve for *n* unknowns). The statistical model (Eq 3) includes two sensitivity coefficients, *α*_*i*_ and *β*_*i*_, so each batch generally needs to include at least two different standard measurements. On the other hand, if one assumes that measurements do not have a consistent offset, meaning that all of the *α*_*i*_ values are assumed to equal zero, then each batch only needs one standard measurement. Fig 1 illustrates this latter situation. Our work also fit this latter situation because we corrected for background fluorescence before starting our data calibration. Any batches that do not include as many standard measurements as unknown sensitivity coefficients need to be removed from the data analysis. In the process, any samples that were only measured in these batches get removed too.

Next, it is helpful to define several variables. Define *N*_*B*_ as the number of batches (number of rows), *N*_*S*_ as the number of samples (number of columns), and *n*_*ij*_ as the number of measurements of sample *j* in batch *i* (number of entries at site *i*,*j*). Generalizing this last definition, *n*_*All*,*j*_ is the total number of measurements of sample *j* (the number of entries in column *j*), *n*_*i*,*All*_ is the total number of measurements in batch *i* (the number of entries in row *i*), and *n*_*All*,*All*_ is the total number of measurements (the number of entries in the table). Also, define *T* as the list of standards; for example, there is one standard in Fig 1, which is sample number 1, so *T* = {1} in that case. Finally, *n*_*i*,*T*_ is the number of standard measurements in batch *i*.

#### (3a) Fit sensitivity coefficients

As the first step of the 2-step method (the calibration stage), a line is fit to the standard data in each batch using least-squares methods. This provides best-fit *a*_*i*_ and *b*_*i*_ values as estimates for the true *α*_*i*_ and *β*_*i*_ sensitivity coefficients. If the *α*_*i*_ sensitivities are not assumed to equal zero, then the *a*_*i*_ and *b*_*i*_ values are found using the standard results for simple linear regression [24],

$$b_{i}=\frac{{\langle x_{j}y_{ijk}\rangle }_{T,k}-{\langle x_{j}\rangle }_{T,k}{\langle y_{ijk}\rangle }_{T,k}}{{\langle x_{j}^{2}\rangle }_{T,k}-{\langle x_{j}\rangle }_{T,k}^{2}}$$ (4)

$$a_{i}={\langle y_{ijk}\rangle }_{T,k}-b_{i}{\langle x_{j}\rangle }_{T,k}.$$ (5)

Angle brackets indicate averaging over the indices that are listed in their subscripts. In this case, the average is over all standards that were measured in any particular batch. For example,

$${\langle x_{j}y_{ijk}\rangle }_{T,k}\equiv \frac{1}{n_{i,T}}\sum_{j\in T}\sum_{k=1}^{n_{ij}}x_{j}y_{ijk}$$ (6)

$${\langle x_{j}^{2}\rangle }_{T,k}\equiv \frac{1}{n_{i,T}}\sum_{j\in T}\sum_{k=1}^{n_{ij}}x_{j}^{2}.$$ (7)

If the *α*_*i*_ sensitivities are assumed to equal zero, then all of the *a*_*i*_ values clearly equal zero and the *b*_*i*_ values simplify to

$$b_{i}=\frac{{\langle x_{j}y_{ijk}\rangle }_{T,k}}{{\langle x_{j}^{2}\rangle }_{T,k}}.$$ (8)

Note that an intuitively sensible, but incorrect, approach would be to compute the *b*_*i*_ values in the latter case by simply solving *y*_*ijk*_ ≈ *b*_*i*_*x*_*j*_ for *b*_*i*_ to give *b*_*i*_ ≈ *y*_*ijk*_/*x*_*j*_ and then averaging these values to give *b*_*i*_ = 〈*y*_*ijk*_/*x*_*j*_〉_*T*,*k*_. Eq 8 is different in that it weights each term in this average by *x*_*j*_^2^. Doing so correctly emphasizes those data points that are likely to have larger measurement values and hence lower relative errors (see the derivations in the appendix).

#### (3b) Compute analyte amounts

In the second step of the 2-step method (the prediction stage), the amount of analyte in each unknown sample is computed by inverting the statistical model equation (Eq 3), while using the *a*_*i*_ and *b*_*i*_ estimates for *α*_*i*_ and *β*_*i*_. Then, averaging results over all analyses of each sample yields the following estimate for the sample’s analyte amount:

$$x_{j}=\frac{{\langle b_{i}y_{ijk}\rangle }_{ik}-{\langle a_{i}b_{i}\rangle }_{ik}}{{\langle b_{i}^{2}\rangle }_{ik}}.$$ (9)

As in Eq 8, this solution is weighted to emphasize the data points that have larger measurement values and hence lower relative errors. In contrast, the intuitively sensible but incorrect approach gives the average as *x*_*j*_ = 〈(*y*_*ijk*_–*a*_*i*_)/*b*_*i*_〉_*ik*_, but this over-emphasizes data points that are likely to have large errors and under-emphasizes those that are likely to have small errors.

#### (4) Compute standard deviations (variability) and standard errors (precision)

Our statistical model assumes that measurements have normally distributed errors. To estimate the standard deviation of those errors, we compute the root mean square (rms) average deviation of the actual measurements, *y*_*ijk*_, away from where we would have expected them, *a*_*i*_+*b*_*i*_*x*_*j*_,

$$\sigma =\sqrt{\frac{1}{n_{All,All}-2N_{B}-N_{S}}\sum_{i=1}^{N_{B}}\sum_{j=1}^{N_{S}}\sum_{k=1}^{n_{ij}}{\left(y_{ijk}-a_{i}-b_{i}x_{j}\right)}^{2}}.$$ (10)

The denominator represents the number of degrees of freedom, which is one for each of the *n*_*All*,*All*_ data points, minus the number of fit coefficients. There are 2*N*_*B*_+*N*_*S*_ fit coefficients if the *α*_*i*_ values are not assumed to equal zero (for the *a*_*i*_, *b*_*i*_, and *x*_*j*_ values), as shown in Eq 10, and *N*_*B*_+*N*_*S*_ if the *α*_*i*_ values are assumed to equal 0. Because we assumed Gaussian distributed noise, about 68% of the measurements should be within one standard deviation of their expected values and about 95% within two standard deviations. Measurements that are many standard deviations away from their expected values are outliers, which may warrant further inspection and possible removal. Importantly though, if the minimum number of standards was measured in each batch, which is typical, then it is impossible to determine if any of those measurements are outliers because the sensitivity parameters were computed directly from them.

Separate standard deviations represent the variability in the different analyte amount estimates, which came from Eq 9. These estimates are weighted means, so their variabilities are computed as weighted standard deviations, for which the general equation is [25]

$$SD=\sqrt{\frac{n\sum_{i}w_{i}{\left(z_{i}-\bar{z}\right)}^{2}}{d\sum_{i}w_{i}}}.$$ (11)

Here, *z*_*i*_ represent the data, *w*_*i*_ represent the weights, $\bar{z}$ is the sample mean, *n* is the number of data points, and *d* is the number of degrees of freedom. Applying this to the sample analyte amounts and simplifying gives

$$SD_{j}=\sqrt{\frac{n_{All,j}}{n_{All,j}-1}\cdot \frac{{\langle {\left(y_{ijk}-a_{i}-b_{i}x_{j}\right)}^{2}\rangle }_{ik}}{{\langle b_{i}^{2}\rangle }_{ik}}}.$$ (12)

The number of degrees of freedom is *n*_*All*,*j*_-1 because there are *n*_*All*,*j*_ terms in the sum but the *x*_*j*_ value was constrained through Eq 9.

The standard errors of the means are typically more useful. They reflect how closely the estimated analyte amounts are likely to represent the true analyte amounts. As usual, they are computed by dividing the standard deviations by the square root of the number of measurements being considered [25]. However, doing so yields a lower bound for the standard error because the standard deviations were computed while assuming that the *a*_*i*_ and *b*_*i*_ values equaled their true values and that the *x*_*j*_ value was the only one that needed to be fit to the data. However, all three of these are estimates, which increases the uncertainty for the analyte amounts. Thus, the standard errors are

$$SE_{j}\geq \frac{SD_{j}}{\sqrt{n_{All,j}}}.$$ (13)

The interpretation is that the difference between each computed *x*_*j*_ value and the true analyte amount for the sample is likely to be a Gaussian distributed random variable with standard deviation equal to *SE*_*j*_. This result does not apply to the standards because their analyte amounts are assumed to be known.

### 1-step method

The 1-step method parallels the 2-step method very closely.

#### (1) Tabulate data

The 1-step method uses the same data table as the 2-step method.

#### (2) Remove orphan measurements

The 1-step method relies on standards less than the 2-step method does, but still requires that each measurement can be related to the standard measurements in some way. More precisely, each batch needs at least as many independent “connections” to standard measurements as there are sensitivity coefficients; a batch is connected to a standard if (*i*) it includes a measurement of that standard or (*ii*) it shares a sample with some other batch that is connected to that standard. We call measurements that cannot be connected to enough standard measurements orphans. These orphan measurements need to be removed from the data analysis, along with the samples and batches to which they belong. The 1-step method uses the same definitions for the *N*_*B*_, *N*_*S*_, *n*_*i*,*j*_, *T*, and other variables as the 2-step method.

#### (3) Iteratively fit sensitivities and analyte amounts

The single step of the 1-step method is to simultaneously fit the *a*_*i*_, *b*_*i*_, and *x*_*j*_ values to the data while assuming the statistical model given in Eq 3. This can be accomplished in many ways, including with deterministic and stochastic minimization algorithms [24]. However, we found that computing the sensitivities and analyte amounts iteratively, using equations derived in the appendix, was particularly simple and efficient. In this method, one first guesses all of the sensitivities. An adequate approach is simply to set all of them to 1 initially, but we found that results converged faster when we guessed as many as possible using Eqs 4, 5 and 8 from the 2-step method and then set the rest to their means. Next, the unknown analyte amounts are computed from

$$x_{j}=\frac{{\langle b_{i}y_{ijk}\rangle }_{ik}-{\langle a_{i}b_{i}\rangle }_{ik}}{{\langle b_{i}^{2}\rangle }_{ik}},$$ (14)

which is identical to Eq 9. Then, the sensitivities are computed from

$$b_{i}=\frac{{\langle x_{j}y_{ijk}\rangle }_{jk}-{\langle x_{j}\rangle }_{jk}{\langle y_{ijk}\rangle }_{jk}}{{\langle x_{j}^{2}\rangle }_{jk}-{\langle x_{j}\rangle }_{jk}^{2}}$$ (15)

$$a_{i}={\langle y_{ijk}\rangle }_{jk}-b_{i}{\langle x_{j}\rangle }_{jk}$$ (16)

if the *α*_*i*_ values are not assumed to equal zero, and

$$b_{i}=\frac{{\langle x_{j}y_{ijk}\rangle }_{jk}}{{\langle x_{j}^{2}\rangle }_{jk}}$$ (17)

if they are. These equations only differ from Eqs 4, 5 and 8 in that they include averages over all measurements in a batch rather than just the standard measurements. Iterating over Eqs 14 to 17 leads to the best-fit values for the analyte amounts and sensitivities. We continued until all sensitivity parameter and analyte amount estimates changed by less than 1 part in 10^5^ between subsequent iterations, which never took more than a few hundred iterations (340 for our immunoblot data and about 70 for most of the validation tests described below).

In the appendix, we show that this iterative approach always converges upon the parameters that produce the best fit between the statistical model (Eq 3) and the data. However, this does not necessarily mean that these parameters are physically reasonable. For example, the model might fit the data best when some analyte values are zero, negative, or extremely large. These unreasonable results can be prevented by defining an allowed range for each parameter and using Eqs 14 to 17 when the respective parameter is within this range, but otherwise fixing the parameter to the nearest range endpoint.

#### (4) Compute standard deviations and standard errors

The standard deviation and standard error equations that are presented above in Eqs 10 to 13 apply here as well. However, the standard deviation *can* be used to identify outlier standard measurements in this case, even if relatively few standard measurements were made, because these sensitivity parameters were computed from all of the measurements instead of just the standard measurements.

### Validation

We validated our method by analyzing artificially generated data sets and comparing the fit results with the true parameters from which the data were generated. To create one of the artificial data sets, we first assumed two standard samples with analyte amounts of 5 and 15. We then generated 18 more random true analyte amounts (Gaussian distributed with mean 10 and standard deviation 3) to represent 18 unknown samples. Next, we generated 20 random *α* and *β* sensitivity values (Gaussian distributed with mean 100 and 10 and standard deviation 30 and 3, respectively) to represent 20 batches. Finally, we generated 400 artificial measurements. For each, we chose a random sample and random batch and then used Eq 3 to compute the measurement value, which included Gaussian-distributed measurement noise (standard deviation 20).

**Fig 2:**
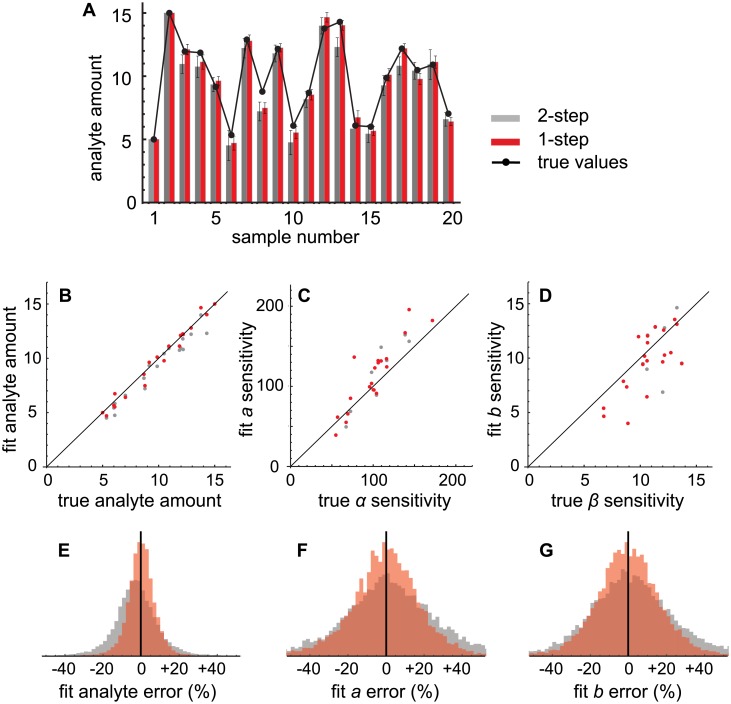
Comparison of the 1-step and 2-step methods using artificial data. A. Sample analyte amounts for an artificial data set. Here and in subsequent panels, black features represent the true analyte amounts, gray features represent results from the 2-step method, and red features represent results from the 1-step method. Error bars represent standard errors. (B-D) Comparison of computed sample analyte amounts, *a* sensitivity coefficients, and *b* sensitivity coefficients with their true values for the same artificial data set. (E-G) Histograms of errors between fit values and true values for computed sample analyte amounts, *a* sensitivity coefficients, and *b* sensitivity coefficients for 1000 artificial data sets. Note that the 1-step method yields more accurate data calibration. See main text for details.

We analyzed these data with the 1-step and 2-step methods. The results, in Fig 2A–2D show that both analysis methods were able to recover the true parameters from the data reasonably well, but the 1-step method estimates were generally closer to the true values. There were enough standard measurements in these data that all analyte amounts could be estimated using both methods. However, only 8 of the batch sensitivities could be estimated using the 2-step method because the other batches had insufficient standards and so needed to be removed from the analysis (note the relatively few gray data points in panels C and D).

Next, we generated 1000 more data sets in exactly the same way and also calibrated those with both methods. We found that, on average, the 1-step method overestimated analyte amounts by 0.1% and the 2-step method underestimated them by 2.6% (Fig 2E). Further tests showed that these offsets arose from the choices of standards, becoming larger when the standard analyte amounts differed more from typical sample analyte amounts. The offsets were usually about a factor of 10 smaller for the 1-step method. Fig 2E also shows that the 1-step method generally computed individual analyte amounts that were closer to the true values: the root mean square (rms) error for the analyte amounts was 9% for the 1-step method and 15% for the 2-step method. Similarly, Fig 2F and 2G show that the 1-step method computed sensitivity parameters that were closer to their true values: rms errors were 20% and 30% for the *a* sensitivity parameter and 18% and 28% for the *b* sensitivity parameter, for the two methods respectively. Thus, the 1-step method consistently estimated all parameters more accurately than the 2-step method. (To enable meaningful comparisons, this analysis only included parameters that were, for both methods, not from orphaned samples or batches and less than 2-fold away from the true values; about 0.07% of analyte estimates, 0.1% of *a* value estimates, and 0.2% of *b* value estimates had greater that 2-fold errors, which would have disproportionately affected results).

We also compared the computed standard errors and standard deviations against the true ones as a way of validating Eqs 13 and 9. The average 1-step and 2-step standard error estimates were 76% and 73% of the actual deviations between the computed and true analyte amounts. These show reasonable agreement and are consistent with the inequality in Eq 13. The average 1-step and 2-step measurement standard deviation estimates were 20.02 and 24.0 units, respectively, while the true value was 20 units, again showing good agreement. This latter result is particularly significant because the standard deviation estimate quantifies the difference between the statistical model and the data. The smaller result for the 1-step method shows that it produced a better fit to the data. Furthermore, the fact that its standard deviation is essentially the same as the true value, which is a theoretical lower limit for the fit standard deviation (on average), implies that the 1-step method produced essentially the best possible results.

Upon further analysis, we found that the 1-step method produced more accurate results for two reasons. First, it included more data points in the calibration due to its decreased dependence on standards (out of the 1000 data sets, none of the batches needed to be removed from the analysis in the 1-step method but 60% of them needed to be removed for the 2-step method). As a result, the 1-step method was able to include more measurements in its averages and hence better reduce the effects of measurement noise. Secondly, the 1-step method computed the sensitivity parameters more accurately, even when there were sufficient standards for both methods, due to other samples that were shared between batches. To investigate these points further, we repeated the validation procedure but used new artificial data sets in which every batch included every standard. As a result, no measurements needed to be removed from either calibration method. In this case, rms errors for the analyte amounts were 8% and 9% for the 1-step and 2-step methods, respectively. The lower value for the 1-step method shows that its consideration of samples that are shared between batches helps it return more accurate results.

We performed several additional validation tests to explore other situations. (*i*) We repeated our validation test of 1000 artificial data sets but with the true values 100-fold larger, 100-fold smaller, and negative, to ensure that the method and our software were equally good with very different parameter values. We found that they worked well and, as before, the 1-step method was consistently more accurate. (*ii*) We generated new validation data using 2-fold higher and 2-fold lower measurement standard deviations to explore the effects of experimental noise. We found that both methods estimated parameters more accurately with lower measurement noise, as expected, but the 1-step method become more accurate more quickly. This showed that the 1-step method does not just improve calibration for experiments with high noise but also improves results for experiments with low noise. (*iii*) We tested the situation in which every batch included the standard but batches did not share any other samples (we assumed that the *α*_*i*_ values were zero, so we only needed one standard per batch; this test used 10 batches, 11 standards, and 100 total measurements). In this case, the 1-step and 2-step methods returned identical results. Subsequent work showed that this is generally true whenever batches have a common standard but no other shared samples. In this case, the average analyte amount rms errors were 15% in both cases. (*iv*) Expanding upon the prior test, we generated data in which every batch included the standard and one additional common sample, but no other shared samples. Now, the 1-step method estimates were more accurate than those from the 2-step method (average analyte rms errors were 9% and 14%). (*iv*) Finally, we generated data in which every batch included every sample. In this case, both methods returned excellent results, each with average analyte amount rms errors of 5.6%. However, the results were not quite the same; the 1-step and 2-step method average sensitivity parameter rms errors were 5% and 12%, respectively, and they estimated the measurement standard deviations as 10 and 14 units, as compared to an actual value of 10 units.

### Protein immunoblot data

**Fig 3:**
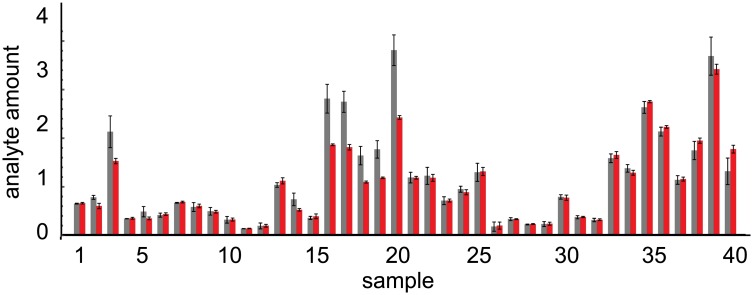
Calibrated experimental immunoblot data. This figure shows calibrated analyte amounts for 40 of our 230 samples that we analyzed with immunoblots. The others were qualitatively similar. Gray bars represent results from the 2-step method, red bars represent results from the 1-step method, and error bars represent standard error values. On average, there were 3.4 calibrated measurements for each sample with the 2-step method and 4.9 for the 1-step method. Note that the 1-step method results have smaller standard errors.

We analyzed our experimental immunoblot data using both methods, of which a small portion of the results are shown in Fig 3. These data are scaled so that the standard (not shown in the figure) has an analyte amount of 1. As part of the analysis, we automatically removed all measurement results that were 4 or more standard deviations away from their expected values, which we deemed to be outliers, and then re-calibrated the remaining data repeatedly until there were no more outliers. This process showed that about 1% of our measurement results were outliers (for comparison, 0.003% would be expected to be more than 4 standard deviations away from the mean if errors were distributed perfectly normally). After all outliers were removed, the 1-step method enabled us to use all of the remaining measurements in the final analysis, whereas we needed to remove about 36% of them with the 2-step method. Results from both methods showed that the calibrated parameters for our data varied quite widely. Sample analyte amounts varied from 0 to about 2, where the standard was defined to have an amount of 1, and sensitivity parameters varied from 26 to about 700, in units of fluorescence units per amount of standard analyte. This latter 27-fold sensitivity range illustrates the wide variability of immunoblotting methods.

Differences between the two methods were more striking with the real data than with the artificial data that we used for validation. Here, the two methods often returned substantially different analyte amount estimates. This did not, of course, indicate which estimates were more accurate because the true analyte amounts were unknown, so the more important difference was that the model (Eq 3) fit the measurement data much better when using parameters found with the 1-step method than with those from the 2-step method. As described above, we observed this by computing the measurement standard deviation, *σ* (Eq 10), which represents the quality of the model fit, or more precisely, the average deviation between the model’s estimate of the true value for each data point and the actual value that was measured for that data point. These standard deviations were 49 fluorescence units for the 1-step method and 82 fluorescence units for the 2-step method (CV of 41% and 68%, respectively).

To better understand these differences, we repeated the 1-step method while only including batches that had standards, so that it would use the exact same data as the 2-step method. This increased the 1-step method standard deviation to 57 fluorescence units. In combination with the prior results, this showed that the 1-step method enabled the model to fit the data better because it includes more data points in the analysis (creating the difference between 49 and 57 fluorescence units) and because it uses information from samples that are replicated over multiple batches in the parameter estimates (creating the difference between 57 and 82 fluorescence units). We are using the 1-step method results for further investigation of these data.

## Discussion

We have described a method for calibrating data to external standards. The conventional approach to calibrating measurement data, which we call the 2-step method, is justifiably nearly ubiquitous. It is simple, intuitive, and convenient. As a result, it can be performed by hand or with spreadsheet software. Also, if there is only a single batch of data, or multiple batches without shared samples, then it is the optimal approach. In this case, it returns the maximum likelihood predictors for the analyte amounts (assuming that the statistical model is correct and that the measurements are weighted properly when averaging, as shown above). However, it does not return the best possible results if the data include samples that are shared among multiple batches because it ignores some of the available information. As a result, the 2-step method is highly sensitive to errors in the standard measurements and is also completely reliant on there being sufficient standards in every batch. On the other hand, our 1-step method uses information from all of the samples that were measured in multiple batches, which decreases its reliance on standards and enables it to return more accurate results. These results are the maximum likelihood predictors, now for the complete data set.

We compared the 1-step and 2-step methods with artificial and real data. Using artificial data, we found that the 2-step method returned analyte amounts that were systematically offset from the true values, whereas those from the 1-step method had essentially no offset. More importantly, analyte amount estimates were typically 50% to 2-fold more accurate with the 1-step method. In addition, the 1-step method invariably returned measurement standard deviation estimates that were essentially the same as the true standard deviations, indicating that it fit the model to the data as well as is theoretically possible. Standard deviation estimates were always larger for the 2-step method. The 1-step method also led to better results with our immunoblotting data. Immunoblotting is inherently imprecise, with large variation between gels and high measurement standard deviations. This presents a problem for quantitative comparisons between samples when not all samples fit on a single gel (batch). In this case, the 1-step method enabled us to estimate protein concentrations from essentially all of our data, including from gels that did not have standards. These estimates are almost certainly more accurate than comparable ones that we calculated using the 2-step method because the 1-step method computed a smaller measurement standard deviation, indicating a better fit between the model and the data.

Throughout most of this work, we have focused on the analyte amount estimates because they are typically of particular interest. However, note that the standard errors of these estimates can be computed as well with minimal additional effort (standard errors indicate the quality of the analyte amount estimate, such that the true analyte value is likely to be within one standard error of the estimate). For this reason, we recommend against the common practice of calibrating multiple runs of an experiment independently and then computing statistics from the calibrated results. This approach throws away valuable information from samples that are shared between batches, does not allow for detection of errors in the standard measurements, and does not improve statistics calculations.

A drawback of the 1-step method is that it requires an iterative computation, making it impractical to perform by hand or in a simple spreadsheet. Nevertheless, this computation is not particularly demanding. Calibrating our immunoblot data set, which comprises 5966 measurements and requires 340 iterations, takes just over 1 minute on a 2013 MacBook laptop computer. From inspection of Eqs 14 to 17, the computational demands scale approximately linearly with the number of measurements, implying that much larger data sets can be calibrated reasonably efficiently as well. A second drawback of the method is that it assumes that instrument or method responses increase linearly with analyte amounts (see Eq 3), which is often not the case. However, it is relatively straightforward to modify the 1-step method as it is presented here to specific non-linear relationships by repeating the derivations presented in the appendix, but for the desired relationship.

The obvious question arises of how to best design experiments so that they yield the most accurate results while calibrating the data with the 1-step method. Although a thorough treatment was beyond the scope of our work, some aspects are reasonably obvious from the design of the method and our validation results. First, standards should be measured in as many of the batches as possible because that minimizes the number of steps that need to be taken to connect unknown sample measurements with standard measurements. Also, it is better to spread replicates of sample measurements out over multiple batches, rather than to perform them all within a single batch, because that improves the ability to cross-calibrate the different batches. Stated differently, it is best to have as many samples as possible in common between the batches, ideally with every sample measured in every batch.

Our software for calibrating data using both the 1-step and 2-step methods is written in Python, is open source, and is in the public domain (i.e. we do not reserve any intellectual property rights). It is available from the supporting information (S1 Text) and online at http://www.smoldyn.org/calibration.html. It can also be used at the same website as an online calibration service.

## Appendix

This appendix derives most of the equations presented above. It is shown at a relatively elementary level to make it widely accessible, so statistics textbooks (e.g. ref. [25]) should be consulted for more rigorous treatments.

From Eq 3, we assume the statistical model

$$y_{ijk}=\alpha_{i}+\beta_{i}x_{j}+\varepsilon_{ijk}.$$ (A.1)

We rearrange the equation and divide both sides by *σ*, the measurement error standard deviation, to yield the scaled measurement errors,

$${\varepsilon^{\prime }}_{ijk}=\frac{\varepsilon_{ijk}}{\sigma }=\frac{y_{ijk}-\alpha_{i}-\beta_{i}x_{j}}{\sigma }.$$ (A.2)

Because we assumed that the measurement noise is Gaussian distributed and independent between data points, the *ε'*_*ijk*_ values are independent normally distributed random variables with zero mean and unit standard deviation. We square both sides of this equation and sum over all data points to yield

$$\sum_{i=1}^{N_{B}}\sum_{j=1}^{N_{S}}\sum_{k=1}^{n_{ij}}{\varepsilon^{\prime }}_{ijk}^{2}=\sum_{i=1}^{N_{B}}\sum_{j=1}^{N_{S}}\sum_{k=1}^{n_{ij}}{\left(\frac{y_{ijk}-\alpha_{i}-\beta_{i}x_{j}}{\sigma }\right)}^{2}.$$ (A.3)

The left side is a sum of squared independent normally distributed random variables, which means that it is itself a random variable and it obeys the chi-squared distribution.

Looking back at Eq A.1, if we knew the exact values of each *α*_*i*_, *β*_*i*_, and *x*_*j*_ but not the *y*_*ijk*_ values, then the assumption that the error is normally distributed with a mean value of zero would imply that the most likely value for *y*_*ijk*_ is the one that arises if the error equals zero. However, we actually know the *y*_*ijk*_ values but not the *α*_*i*_, *β*_*i*_, or *x*_*j*_ values. So, we rearrange the prior statement to claim that the most likely values of *α*_*i*_, *β*_*i*_, and *x*_*j*_, given the known *y*_*ijk*_ values, are those that minimize the computed errors (Eq A.2). This rearrangement is not completely legitimate but is the central ansatz of maximum likelihood estimation and is partially justified by Bayesian analysis [24]. Without going further into the details, we perform maximum likelihood estimation by replacing the true sensitivity coefficients, *α*_*i*_ and *β*_*i*_, in Eq A.3 with the unknown *a*_*i*_ and *b*_*i*_ estimated sensitivity coefficients to yield the following “goodness-of-fit” function,

$$\chi^{2}=\sum_{i=1}^{N_{B}}\sum_{j=1}^{N_{S}}\sum_{k=1}^{n_{ij}}{\left(\frac{y_{ijk}-a_{i}-b_{i}x_{j}}{\sigma }\right)}^{2}.$$ (A.4)

We then minimize this function with respect to each *a*_*i*_, *b*_*i*_, and unknown *x*_*j*_ parameter to find their most likely values. The parameter values that minimize the *χ*^2^ function are called the maximum likelihood predictors because they are the most probable values, within the assumptions of the model.

We find the minimum of *χ*^2^ with respect to *x*_*j'*_, where *j'* is the index of a specific unknown sample, by differentiating *χ*^2^ with respect to *x*_*j'*_ and setting the result to zero:

$$\begin{array}{ll} \frac{\partial \chi^{2}}{\partial x_{j^{\prime }}} & =\frac{\partial }{\partial x_{j^{\prime }}}\sum_{i=1}^{N_{B}}\sum_{j=1}^{N_{S}}\sum_{k=1}^{n_{ij}}{\left(\frac{y_{ijk}-a_{i}-b_{i}x_{j}}{\sigma }\right)}^{2}\\ & =\frac{\partial }{\partial x_{j^{\prime }}}\sum_{i=1}^{N_{B}}\sum_{k=1}^{n_{ij^{\prime }}}{\left(\frac{y_{ij^{\prime }k}-a_{i}-b_{i}x_{j^{\prime }}}{\sigma }\right)}^{2}\\ & =\sum_{i=1}^{N_{B}}\sum_{k=1}^{n_{ij^{\prime }}}2\left(\frac{y_{ij^{\prime }k}-a_{i}-b_{i}x_{j^{\prime }}}{\sigma }\right)\cdot -\frac{b_{i}}{\sigma }\\ & =-\frac{2}{\sigma^{2}}\sum_{i=1}^{N_{B}}\sum_{k=1}^{n_{ij^{\prime }}}b_{i}\left(y_{ij^{\prime }k}-a_{i}\right)+\frac{2}{\sigma^{2}}x_{j^{\prime }}\sum_{i=1}^{N_{B}}\sum_{k=1}^{n_{ij^{\prime }}}b_{i}^{2}. \end{array}$$ (A.5)

Setting the result to zero, renaming *j'* to *j*, and simplifying yields

$$0={\langle b_{i}y_{ijk}\rangle }_{ik}-{\langle a_{i}b_{i}\rangle }_{ik}-x_{j}{\langle b_{i}^{2}\rangle }_{ik}.$$ (A.6)

This result represents one equation for each unknown sample. Minimizing *χ*^2^ with respect to *a*_*i*_ and *b*_*i*_ are analogous, yielding

$$0={\langle y_{ijk}\rangle }_{jk}-b_{i}{\langle x_{j}\rangle }_{jk}-a_{i}$$ (A.7)

$$0={\langle x_{j}y_{ijk}\rangle }_{jk}-a_{i}{\langle x_{j}\rangle }_{jk}-b_{i}{\langle x_{j}^{2}\rangle }_{jk}.$$ (A.8)

These results represent one pair of equations for each batch. In principle, Eqs A.6 to A.8 can be solved for the unknown *a*_*i*_, and *b*_*i*_, and *x*_*j*_ values. However, this appears to be analytically intractable so instead we rearrange them to yield

$$x_{j}=\frac{{\langle b_{i}y_{ijk}\rangle }_{ik}-{\langle a_{i}b_{i}\rangle }_{ik}}{{\langle b_{i}^{2}\rangle }_{ik}}$$ (A.9)

$$b_{i}=\frac{{\langle x_{j}y_{ijk}\rangle }_{jk}-{\langle x_{j}\rangle }_{jk}{\langle y_{ijk}\rangle }_{jk}}{{\langle x_{j}^{2}\rangle }_{jk}-{\langle x_{j}\rangle }_{jk}^{2}}.$$ (A.10)

$$a_{i}={\langle y_{ijk}\rangle }_{jk}-b_{i}{\langle x_{j}\rangle }_{jk}$$ (A.11)

If it is assumed that the *α*_*i*_ values all equal zero, then the *a*_*i*_ values are set to zero and the solutions for *x*_*j*_ and *b*_*i*_ get simplified to

$$x_{j}=\frac{{\langle b_{i}y_{ijk}\rangle }_{ik}}{{\langle b_{i}^{2}\rangle }_{ik}}$$ (A.12)

$$b_{i}=\frac{{\langle x_{j}y_{ijk}\rangle }_{jk}}{{\langle x_{j}^{2}\rangle }_{jk}}$$ (A.13)

These equations cannot be computed sequentially because each equation requires knowledge of the other results. Thus, the approach taken by the 2-step method is to limit the averages in Eqs A.10, A.11 and A.13 to just those samples which have known analyte amounts, which are the standards. After this, Eqs A.9 or A.12 can be computed without problems. Alternatively, the approach taken by the 1-step method is to compute the equations iteratively, which then yields the best-fit *a*_*i*_, *b*_*i*_ and *x*_*j*_ values.

This raises the question of whether the iterative solution is certain to converge to the global minimum *χ*^2^ value, or whether it might converge to a different local minimum or even fail to converge altogether. To answer this, we observe first that the *χ*^2^ function, Eq A.4, is a quadratic function of each unknown parameter with non-negative curvature (e.g. in Eq A.5, the slope of the first derivative of *χ*^2^ with respect to *x*_*j*_ is 2〈*b*_*i*_^2^〉_*ik*_/*σ*^2^, which is necessarily non-negative). This implies that *χ*^2^ only has a single minimum, which is the global minimum. We also observe that the *χ*^2^ function is everywhere non-negative, from Eq A.4, and that each iteration is certain to either reduce or maintain the prior *χ*^2^ value. This latter argument follows because Eqs A.9 to A.13 each identify the value that minimizes *χ*^2^ for its particular parameter, so each time one of them changes a parameter, it always reduces *χ*^2^. Because *χ*^2^ cannot decrease indefinitely, this implies that the iterative procedure must converge. Finally, every parameter gets optimized, so the iterative procedure must converge at the global *χ*^2^ minimum, as desired. Note however, that the *χ*^2^ function may have zero curvature on some parameters, causing the global minimum to be not a single point in parameter space but a line or larger region. This would arise from insufficient data points and hence an underdetermined system of equations. In this case, which also arises in the 2-step method, the prior arguments showed that the iterative procedure will return one set parameters that represent the *χ*^2^ minimum, but there will also be other parameter combinations that are equally good. To further convince ourselves that the iterative method leads to the correct solutions, we also minimized *χ*^2^ using Mathematica’s “NMinimize” function for a series of validation data sets. In all cases, results were identical but the iterative approach was many-fold faster.

To compute the measurement standard deviation, we start with the fact that the mean of a chi-squared distribution is equal to the number of random variables that are summed. In Eq A.4, the *χ*^2^ sum includes *n*_*All*,*All*_ terms, suggesting that this would be the mean of the distribution. However, we don’t know the true *α*_*i*_, *β*_*i*_, or *x*_*j*_ values, but only those that we fit by minimizing *χ*^2^, which reduces the mean by 2*N*_*B*_+*N*_*S*_ degrees of freedom. Using the assumption that any specific data set is likely to be reasonably typical, we equate *χ*^2^ to *n*_*All*,*All*_–2*N*_*B*_–*N*_*S*_, yielding

$$\chi^{2}=\sum_{i=1}^{N_{B}}\sum_{j=1}^{N_{S}}\sum_{k=1}^{n_{ij}}{\left(\frac{y_{ijk}-a_{i}-b_{i}x_{j}}{\sigma }\right)}^{2}=n_{All,All}-2N_{B}-N_{S}.$$ (A.14)

Solving for the measurement standard deviation then yields

$$\sigma =\sqrt{\frac{1}{n_{All,All}-2N_{B}-N_{S}}\sum_{i=1}^{N_{B}}\sum_{j=1}^{N_{S}}\sum_{k=1}^{n_{ij}}{\left(y_{ijk}-\alpha_{i}-\beta_{i}x_{j}\right)}^{2}}$$ (A.15)

Finally, we solve for the individual sensitivity coefficient and analyte standard deviations. Both are simply weighted averages, so we use the general equations for a weighted standard deviation (main text Eq 11) to yield the results

$$SD_{x_{j}}=\sqrt{\frac{n_{All,j}}{n_{All,j}-1}\cdot \frac{{\langle {\left(y_{ijk}-a_{i}-b_{i}x_{j}\right)}^{2}\rangle }_{ik}}{{\langle b_{i}^{2}\rangle }_{ik}}}$$ (A.16)

$$SD_{a_{i}}=\sqrt{\frac{n_{i,All}}{n_{i,All}-1}\langle {\left(y_{ijk}-b_{i}x_{j}-a_{i}\right)}^{2}\rangle }$$ (A.17)

$$SD_{b_{i}}=\sqrt{\frac{n_{i,All}}{n_{i,All}-1}\cdot \frac{{\langle {\left(y_{ijk}-a_{i}-b_{i}x_{j}\right)}^{2}\rangle }_{jk}}{{\langle x_{j}^{2}\rangle }_{jk}}}$$ (A.18)

Dividing these results by the square root of the number of data points yields estimates for the standard errors.

## Supporting Information

S1 Text Program for generating and analyzing data. This Python program analyzes data using the 1-step and 2-step calibration methods. It can import the data from files or generate synthetic data using the statistical model in Eq 3. It is identical to the online software at http://www.smoldyn.org/calibration.html. It is open source and in the public domain.
(PY) Click here for additional data file.
